# Linked vaccine adverse event data from VAERS for biomedical data analysis and longitudinal studies

**DOI:** 10.1186/s13040-014-0036-y

**Published:** 2014-12-31

**Authors:** Cui Tao, Puqiang Wu, Yi Luo, Yuji Zhang

**Affiliations:** School of Biomedical Informatics, The University of Texas Health Science Center at Houston, Houston, TX USA; University of Wisconsin-Madison, Madison, WI USA; Department of Computer Science and Engineering, Lehigh University, Bethlehem, PA USA; Division of Biostatistics and Bioinformatics, University of Maryland Greenebaum Cancer Center, Baltimore, MD USA; Department of Epidemiology and Public Health, University of Maryland School of Medicine, Baltimore, MD USA

## Abstract

**Background:**

Vaccines have been one of the most successful public health interventions to date. The use of vaccination, however, sometimes comes with possible adverse events. The U.S. FDA/CDC Vaccine Adverse Event Reporting System (VAERS) currently contains more than 200,000 reports for post-vaccination events that occur after the administration of vaccines licensed in the United States. Although the data from the VAERS has been applied to many public health and vaccine safety studies, each individual report does not necessarily indicate a casuality relationship between the vaccine and the reported symptoms. Further statistical analysis and summarization needs to be done before this data can be leveraged.

**Methods:**

This paper introduces our efforts on representing the vaccine-symptom correlations and their corresponding meta-information extracted from the VAERS database using Resource Description Framework (RDF). Numbers of occurrences of vaccine-symptom pairs reported to the VAERS were summarized with corresponding proportional reporting ratios (PRR) calculated. All the data was stored in an RDF file. We then applied network analysis approaches to the RDF data to illustrate a use case of the data for longititual studies. We further dicussed our vision on integrating the data with vaccine information from other sources using RDF linked approach to facilitate more comprehensive analyses.

**Results:**

The 1990–2013 data from VAERS has been extracted from the VAERS database. There are 83,148 unique vaccine-symptom pairs with 75 vaccine types and 5,865 different reported symptoms. The yearly and over PRR values for each reported vaccine-symptom pair were calculated. The network properties of networks consisting of significant vaccine-symptom associations (i.e., PRR larger than 1) were then investigated. The results indicated that vaccine-symptom association network is a dense network, with any given node connected to all other nodes through an average of approximately two other nodes and a maximum of five nodes.

**Electronic supplementary material:**

The online version of this article (doi:10.1186/s13040-014-0036-y) contains supplementary material, which is available to authorized users.

## Background

Vaccines have been one of the most successful public health interventions to date with most vaccine-preventable diseases having declined in the United States by at least 95-99%. However, vaccines are pharmaceutical products that carry risks. They interact with the human immune systems and could permanently alter gene molecular structures. “Under the National Childhood Vaccine Injury Act of 1986, over $2 billion has been awarded to children and adults for whom the risks of vaccine injury were 100%” [1]. Potential relationships between vaccines and particular vaccine adverse events (VAE) may exist, but not well studied yet. The U.S. FDA/CDC Vaccine Adverse Event Reporting System (VAERS) is a national vaccine safety surveillance program for post-vaccination adverse events (AE) that occur after the administration of vaccines licensed in the United States [2]. Currently the VAERS contains more than 200,000 reports in total. Patients or healthcare providers submit reports about cases of adverse events they have experienced on the VAERS website by providing information ranging from vaccine type, gender, age, symptoms and detailed description of occurred symptoms to onset dates, life-threatening status, hospitalization status, and death-status. The objectives of VAERS are to detect new, unusual, or rare vaccine adverse events; determine patient risk factors for particular types of adverse events; identify vaccine lots with increased numbers or types of reported adverse events; and assess the safety of newly licensed vaccines [2].

Although a report was submitted into the VAERS system, that by no means is an absolute declaration that the vaccine had direct correlation with the reported symptoms. The causality relationship between a vaccine and an adverse event cannot be simply assumed by the VAERS report. In this study, we do not only focus on the raw data from the VAERS system, but also the correlation of vaccines and symptoms. Through statistical analysis, the correlation can be better accessed by relating frequency of a specific symptom to the corresponding vaccine and the related symptom with all the vaccines in the system. We represent information obtained and summarized from the VAERS database in the Resource Description Framework (RDF) format to facilitate further integration with other vaccine relevant data for more comprehensive analysis. Armed with such knowledge, the ability to predict adverse events, or to design new vaccine approaches that minimize or eliminate serious vaccine-related reactions could be devised, consistent with a more personalized or individual approach to vaccine practice. In the following sections of this paper, we use “symptom” and “adverse event” in a interchangeable manner.

After possible vaccine-adverse event correlations are identified, how to organize these high-dimensional correlation data and facilitate pattern recognition by clinical researchers is still a big challenge. In recent years, network analysis emerges as a very promising approach to address this. Network analysis allows simultaneous representation of complex associations (e.g., protein-protein interactions) among key elements (e.g., gene or proteins) in a system (e.g., gene regulatory networks). For example in the social networks, the nodes are individuals, organizations, or even the entire societies, and the edges are social relationships between the nodes. During last two decades, network-based computational approaches gained popularity and have become a new paradigm to investigate associations among biological entities (e.g., drugs, diseases, and genes). Applications of these approaches include drug repositioning [3,4], disease gene prioritization [5-7], and identification of disease relationships [8,9]. These network analysis approaches are usually developed based on the observations from real-world networks. First, most real-world networks (e.g., WWW network, protein-protein interaction network, and social network) are not randomly organized but are driven by preferential attachment and growth (e.g., some nodes have more connections than others). Such networks are called “scale-free” networks. In the “scale-free” network, the most highly connected nodes are called “hub’ nodes. Second, most real world networks are modular, comprised of small, densely connected groups of nodes. Network analysis metrics and algorithms have been designed to identify network hub nodes and modules in a scale-free network. For instance, in our previous work, we developed a network analysis approach to identify vaccine-related networks and their underlying structural information from PubMed literature abstracts, which were consistent with that captured by the Vaccine Ontology (VO) [10]. The modular structure and hub nodes of these vaccine networks reveal important unidentified knowledge critical to biomedical research and public health and to generate testable hypotheses for future experimental verification.

The rest of the paper is organized as follows. In Section 2, we discuss our methodology on data collection, summarization, representation, and analysis. In Section 3, we discuss the result of our preliminary study. In Section 4, we introduce our vision on further integrating the VAERS data with more vaccine data sources. Finally in Section 5, we conclude the paper and discuss future directions.

## Methods

### The VAERS data preparation

All of the VAERS data was downloaded from the reporting system’s website (http://vaers.hhs.gov/index). The necessary files from 1990 to 2013 were then loaded into a MySQL relational database. More specifically, three tables are included in the database: Data, Vaccine, and Symptom. The Data table contains information including VAERS ID, date the report was received, the state patient was in, age of patient, sex, and detailed description of the symptom (e.g., if the symptom was life threatening, if the patient in the report died and if-so the date of death, if the patient ever attend the ER for treatment, and if so, how many days was the patient administered at the hospital.) The Vaccine table includes information about the vaccine administered to the patient such as vaccine manufacturer, type of vaccine, dosage of the vaccine, vaccination route, vaccination site, and vaccination name. Vaccine types are annotated with Vaccine Code (https://vaers.hhs.gov/glossary/). The Symptom table contains a list of symptom terms (MedDRA terms) involved in the report. Completed information about one report can be jointed from the three tables using VAERS ID.

### The VAERS data summarization

As we discussed before, the VAERS is a spontaneous reporting system which contains unverified reports with inconsistent data quality. Symptoms reported occurring after vaccination do not necessarily have a causality association with the vaccine. In addition to the raw data downloaded from VAERS, we also used statistical methods to summarize meta-level features of vaccine-symptom pairs. For each vaccine-symptom pair, we calculated the following features (1) each year (from 1990–2013) the number of reports that contains the pair; (2) the distribution of reports by gender each year (3) the distribution of reports by age groups; and (4) overall proportional reporting ratio (PRR) and yearly PRRs [11]. A PRR is the ratio between the frequency with which a specific symptom (adverse event) occurs for a vaccine of interest (relative to all symptoms reported for the vaccine) and the frequency with which the same symptom occurs for all vaccines reported to the VAERS (relative to all symptoms for all vaccines reported to VAERS) [12]. A yearly PRR is calcuated using the data only for one particular year (e.g., reports for year 2013). A PRR greater than 1 suggests that the post-vaccination symptom (adverse event) is more commonly observed for individuals administrated with the particular vaccine, relative to all other vaccines reported to the VAERS.

### RDF representation

We represented vaccine-symptom pairs as well as the summarization features in Resource Description Framework (RDF). RDF is a W3C standard that specifies a graph-based data model for representing data. Each piece of information is represented as a triple: subject, predicate and object. The RDF representations will allow efficient querying and visualization of relationships between important biomedical entities. A distinguishing characteristic of RDF and ontologies compared to the conventional relational database is “their degree of connectedness, their ability to model coherent, linked relationships” [13]. After representing the associations using RDF graphs, it will enable us to leverage existing Semantic Web tools to explore the Semantic Web Linked Data in a flexible and scalable way. Moreover, it will enable powerful data integration among heterogeneous data sets, which is a well-known challenge in the translational science study community.

In order to present the information of vaccine-symptom associations, we first created meta-level classes and properties. OWL classes have been created to represent VaccineSymotomAssociation, Vaccine, and Symptom. OWL object properties such as hasSymptom, hasVaccine, hasOVerallPRR, hasPRRDetail, hasVAERSAgeDetail, and hasVAERSGenderDetail have been created to represent the meta information of a vaccine-symptom pair. OWL data properties such as hasOverallPRR, hasYear, hasAgeGroup, hasGenderGroup, hasCount, and hasPercentage have been generated to represent the values of the data. Once the meta-level information has been defined, we can use the defined classes to specify the semantic type of the entities in the VAERS data set, and use the defined properties to specify the relations between entities and their associated data values. The text below shows a partial representation of a vaccine symptom pair in RDF. We first created a new OWL instance for the pair itself (VaccineSymptomMeta_39777), we then appended more information for this instance. Lines 2 and 3 defines the symptom and the vaccine involved in this association respectively. Line 4 shows the value of the overall PRR. Lines 5–7 shows the detailed information for the PRR value of this pair in year 2009. Lines 8–12 shows the information about this vaccine-symptom association for a specific age group (less then one year old) in year 2009. Lines 13–17 shows the detailed information about this vaccine-symptom association for the male gender in 2009. Information for other years, age groups, and gender groups can be represented similarly. With this RDF representation, we can easily append new information about an association. For example, we can add new detailed information extracted from VAERS when new data is avaliable. The current version if the data can be downloaded from: https://sbmi.uth.edu/ontology/files/vaersRDF.ttl

1. vaers:VaccineSymptomMeta_39777
2. vaers:hasSymptom vaers:intussusception;
3. vaers:hasVaccine vaers:PNC;
4. vaers:hasOverallPRR “4.7518”^^xsd:float;
5. vaers:hasPRRDetail [
6. vaers:hasPRR “7.1539”^^xsd:float;
7. vaers:hasYear “2009”^^xsd:long];
8. vaers:hasVAERSAgeDetail [
9. vaers:hasAgeGroup “LESS_THAN_ONE”^^xsd:string;
10. vaers:hasCount “63”^^xsd:long;
11. vaers:hasPercentage “1.0”^^xsd:float;
12. vaers:hasYear “2009”^^xsd:long];
13. vaers:hasVAERSGenderDetail [
14. vaers:hasCount “31”^^xsd:long;
15. vaers:hasGenderGroup “MALE”^^xsd:string;
16. vaers:hasPercentage “0.4920635”^^xsd:float;
17. vaers:hasYear “2009”^^xsd:long ];

### Network analysis

The network analysis and visualization was performed in the Cytoscape tool [14]. Cytoscape is an open-source platform for integration, visualization, and analysis of biological networks. Its functionalities can be extended through Cytoscape plugins. Scientists from different research fields have contributed more than 160 useful plugins so far. These comprehensive features allow us to perform thorough network-level analyses, visualization of our association tables, and integration with other biological networks in the future. We used NetworkAnalyzer plugin (http://med.bioinf.mpi-inf.mpg.de/netanalyzer//index.php) to calculate average node degree, average path length, and network diameter for each vaccine-adverse event network generated from VAERS.

## Results

Overall, we have extracted 2,346,367 pairs of vaccine-symptom combinations from the VAERS system, with 83,148 distinct pairs. Among all these vaccines and adverse events reposted in the VAERS, we identified 53,742 vaccine-adverse event associations with overall PPR ratio greater than 1 using all reports submitted to VAERS between 1990 and 2013 (data not shown). Over a 23-year period, 75 different vaccines and 5,865 different adverse events were identified to have 191,027 significant associations, i.e., associations reported significant in at least one year report in the system. For the network consisting of associations with overall significant PPR ratios, the average shortest path and the network diameter were 2.48 and 5, respectively (Table 1). This demonstrates that the vaccine-adverse event network is dense, with any given node connected to all other nodes through an average of approximately two other nodes and a maximum of five nodes. This is explained partly that many vaccines are coadministered. However, given that there are more adverse events than vaccines in the network, it is plausible that many adverse events were reported together.

**Table 1:** **General characteristics of the networks**

	**N**_**node**_	**N**_**link**_	**Average degree**	**Average path length**	**Network diameter**
**Overall**	5,938	53,742	18.10	2.48	5
**1990**	368	1,312	7.13	2.71	6
**2000**	1,067	5,709	10.7	2.59	5
**2010**	2,641	18,042	13.66	2.62	5

**Figure 1:**
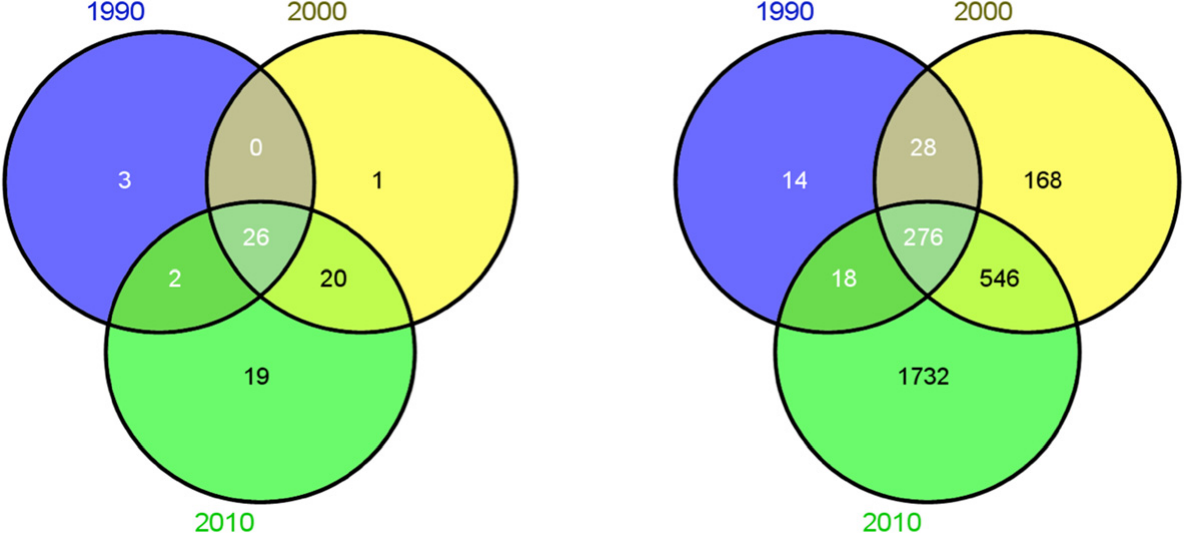
**Venn diagram of nodes among 1990, 2000, and 2010 (left) vaccine nodes; (right) adverse event nodes.**

We further investigated how the vaccine-adverse event network evolves during three decades. Additional files 1, 2 and 3 contains the vaccine-adverse event networks extracted from VAERS reports submitted in 1990, 2000, and 2010. The overall network properties of three networks were shown in Table 1. Among three networks, 14 nodes were in all three networks, including 6 vaccines and 8 adverse events (Figure 1). Haemophilus B Polysaccharide Vaccine (HBPV) is used for a routine immunization of children 24 months to 5 years of age. In three years we investigated, HBPV was associated with different adverse events with significant PPR ratios, suggesting that HBPV may cause different adverse events in different years. This could be due to the difference how this vaccine was manufactured in these years. Such network analysis can help domain experts easily identify such differences and design experiments to further investigate the underlying biological mechanisms. The detailed information of Figure 1 is presented in Additional file 1.

## Linking with other resources

**Figure 2:**
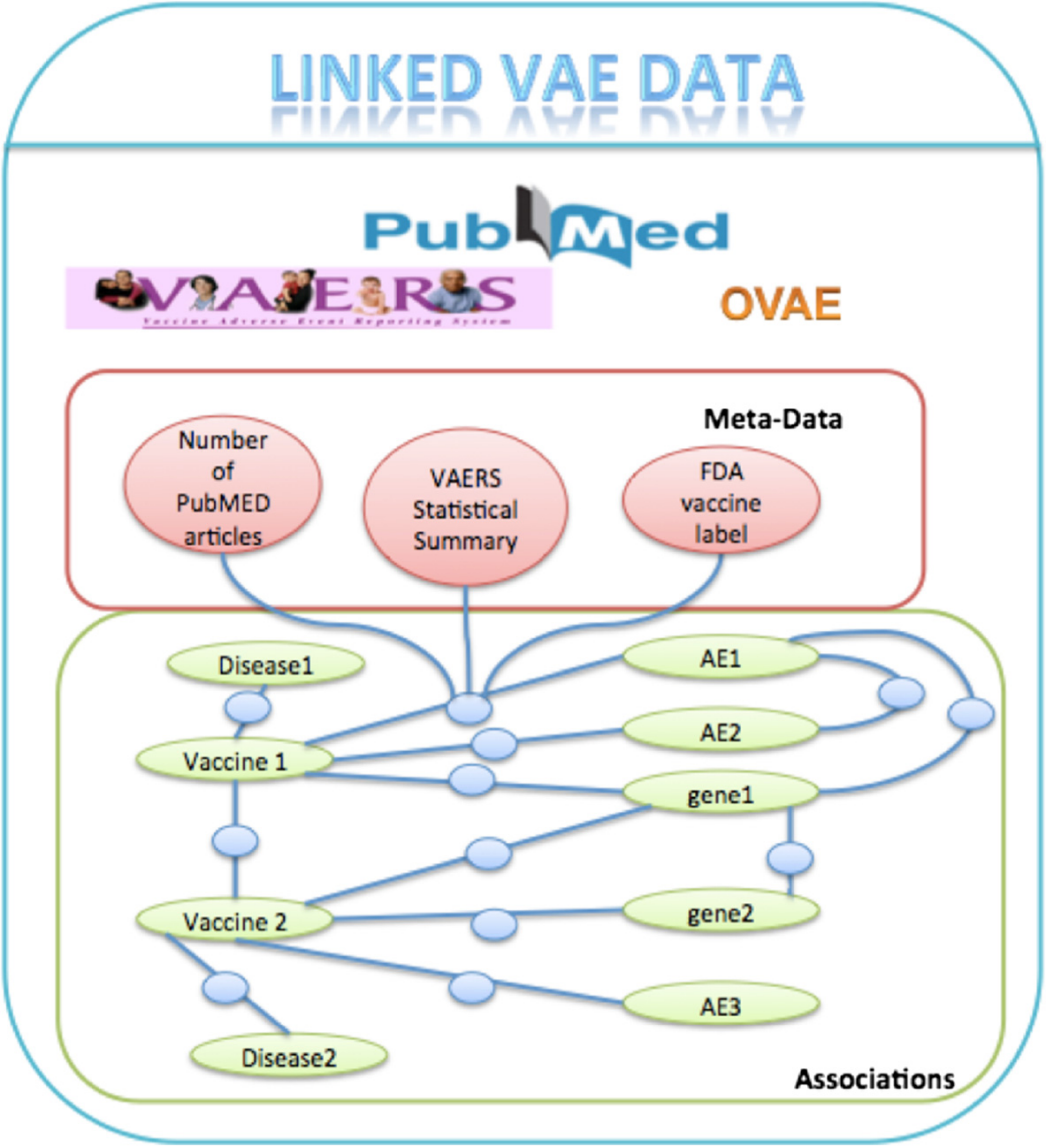
**Linked VAE data overview.**

One unique benefit of RDF representation is that it provides a flexible way to link data from different sources together. With current technologic advances such as high throughput sequencing, transcriptomics, epigenetics, and proteomics, there are big amount of amount of data available for better understanding associations and mechanisms of VAEs and immunogenicity. With the RDF representation, we can integrate the VAERS data with data from other sources such as PubMed literature, Vaccine Label data, and Vaccine ontology to create a Linked VAE data repository. Figure 2 shows the overview. For PubMed data, we have created the SemMed-RDF repository for representing associations among genetic factors, diseases, and drugs extracted from PubMED abstracts [15] based on the Semantic MEDLINE database [16]. This knowledgebase currently contains 843 k disease-disease, 111 k disease-gene, 1277 k disease-drug, 248 k drug-gene, 1900 k drug-drug, and 49 k gene-gene associations, annotated with their provenance information. We have the identified vaccine relevant associations with diseases, symptoms, and genes from SemMed-RDF [10]. This data can be integrated with the VAERS RDF data. In addition, we can also link vaccine relevant information from publicly available ontologies such as Vaccine Ontology (VO) [17] and the Ontology of Vaccine Adverse Event (OVAE) [18]. VO has modeled and classified various vaccines, including all licensed vaccines used in the USA. For each licensed vaccine, VO includes vaccine name, disease or pathogen name, manufacturer, CDC CVX (Codes for Vaccine Administered), host species (e.g., human), vaccine type based on preparation (e.g., killed or inactivated vaccine), vaccine antigen component, and vaccination route). The hierarchy structure of the vaccine in VO classifies the vaccine type based on pathogen taxonomy. OVAE is an ontology that represents and classifies the adverse events recorded in package insert (vaccine label) documents of commercial vaccines licensed by the USA Food and Drug Administration (FDA). Combined these sources, we can create the linked VAE Data, a centralized comprehensive knowledgebase for vaccines and their associations with genetic factors, diseases, and AE can be generated for large-scale computational studies of VAE mechanisms.

## Discussion, future directions, and conclusion

PRR is not the only data mining methods for identifying significant association between vaccines and post-vaccination symptoms [12]. The PRR value > 1 is not an indication that the pair has a causal relationship. For example if a symptom only appeared once for one vaccine type, but not for any other vaccine types, the PRR would be a relatively large number. Given it only happened once, however, it could be a coincidence. Therefore we may need to add threshold to the PRR values or the number of occurrences to filter out this kind of extreme situations. We may also want to add other statistical indicators beside PRR to faciliate further analysis. In addition, more advanced network approaches could be applied to identify underlying associations among vaccines and adverse events, such as subnetwork analysis and network alignments among different populations.

There are a few future directions we plan to pursue: (1) identification of network modules in the vaccine-adverse event network; (2) investigation of vaccine-vaccine associations by bipartite network projection strategy; (3) incorporation of more vaccine-disease association databases (e.g., Semantic MEDLINE database, Vaccine Adverse Event Ontology) to construct more complete vaccine-related networks. Also in this study, we focused on comparing the overall network properties of the vaccine-adverse event association networks generated by different years. In the future, we plan to explore such differences using more advanced network-based computational approaches at different network level, such as subnetwork level and single association level.

In summary, we discussed our effort on representing data summarized from VAERS database using RDF. We then applied network analysis on top of the data to illustrate how network-based analysis can be applied to identify underlying association patterns among vaccines and adverse events.

## Additional files

Additional file 1:
**Vaccine-adverse event network reconstructed from extracted from VAERS reports in 1990.** Green rectangle: vaccine; yellow vee: adverse event. Edge: vaccine-adverse event association with PRR ratio greater than 1. Edge width denotes the PRR ratio of the edge. Additional file 2:
**Vaccine-adverse event network reconstructed from extracted from VAERS reports in 2000.** Green rectangle: vaccine; yellow vee: adverse event. Edge: vaccine-adverse event association with PRR ratio greater than 1. Edge width denotes the PRR ratio of the edge. Additional file 3:
**Vaccine-adverse event network reconstructed from extracted from VAERS reports in 2010.** Green rectangle: vaccine; yellow vee: adverse event. Edge: vaccine-adverse event association with PRR ratio greater than 1. Edge width denotes the PRR ratio of the edge.
